# Comparison of quartz vials with polypropylene vials for rapid cryopreservation of human ovarian tissue

**DOI:** 10.1186/s13048-016-0268-1

**Published:** 2016-09-26

**Authors:** D. Scheiner, G. Bracone, P. Imesch, D. Fink, J. Hehl, B. Imthurn

**Affiliations:** 1 Department of Gynecology, University Hospital Zurich, Frauenklinikstrasse 10, 8091 Zurich, Switzerland; 2 Kantonsspital Luzern, Neue Frauenklinik - Kinderwunsch Zentrum, Andrologie-IVF Labor, Spitalstrasse 2, 6000 Luzern, Switzerland; 3 LMSC-Light Microscopy and Sreening Centre, ETH Zurich, Schafmattstrasse 18, 8093 Zurich, Switzerland; 4 Division of Reproductive Endocrinology, University Hospital Zurich, Frauenklinikstrasse 10, 8091 Zurich, Switzerland

**Keywords:** Rapid cryopreservation, Human ovarian tissue, Quartz vial, HSP70, Bcl2, Ki67

## Abstract

**Background:**

Because higher survival of follicles during the freezing/thawing procedure improves the quality of cryopreserved tissue reimplanted after oncological therapies, defining an optimal method for human ovarian tissue cryopreservation remains a major issue in this field. One option to improve the cryopreservation procedure is to use better materials, i.e., vials with better conductivity. The aim of this study was to compare polypropylene (PP) with quartz vials. Between September 2012 and January 2013, eight patients were recruited. The ovarian cortex was cut into 3 slices, assigned randomly to a fresh and a cryopreserved group in PP (method B) or quartz vials (method C). Histological and immunohistochemical (IHC) analysis were used. For IHC three antibodies were analyzed: Ki67 (proliferation index), Bcl2 (anti apoptotic index) and Hsp70 (stress index).

**Results:**

The majority of GCs showed positive staining for Bcl2 in both cryopreservation device, with higher expression in group C than in group B. Oocytes and their nuclei showed intense positive staining for ki67 in both methods B and C, and also a patch positive stromal cells staining for Ki67. Expression of hsp70 was not increased after cryopreservation.

**Conclusions:**

Cryopreservation using quartz vials led to larger numbers of good follicles while maintaining consistent preservation for stromal cells and vessels.

## Background

Worldwide, at least 60 babies have been born after reimplantation of cryopreserved human ovarian tissue [1–4]. However, tissue cryopreservation is more complex than single cell cryopreservation, as tissues are composed of different types of cells with different water composition. Thus, finding a general protocol suitable for cryopreservation of ovarian tissues is challenging. Several freezing/thawing procedures that differ in dehydration and hydration time, cryoprotectants or protein source have been developed using different devices. Cryopreservation and ischemia after transplantation seem to cause follicle loss; however, the latter can be avoided by using an appropriate micro-surgical technique [5]. Because higher survival of follicles during the freezing/thawing procedure improves fertility, defining an optimal method for cryopreservation remains a major issue in this field. Here, we investigated a rapid freeze/thaw procedure combining the positive aspects of both slow freezing and vitrification. Each of the latter methods has its limitations. S*low freezing* (an equilibrium method) is a long (>3 h) and expensive procedure. It requires significant amounts of liquid nitrogen and a programmable freezing machine, and current methods still cannot preserve all cortical tissue cells. In addition, slow freezing protocols are reported to negatively affect stromal cells, which are also important for follicle dynamics. On the other hand, v*itrification* (a non-equilibrium method) requires very fast cooling rates using a high concentration of cryoprotectants (more than 4 M), which are generally toxic to cells.

The concentration of cryoprotectants as well as the exposure time are important factors in improving follicle cell survival [6]. Another option to improve the cryopreservation procedure is to use better materials, i.e., vials with better conductivity. Compared with polypropylene (PP) vials, quartz vials show better conductivity [7, 8]. The aim of this study was to compare PP with quartz vials and to develop a protocol for improving the survival of follicles and stromal cells in cryopreserved ovarian tissue.

## Methods

Between September 2012 and January 2013, eight patients were recruited at the Department of Gynecology, University Hospital of Zurich. Participation was voluntary, and written informed consent was obtained before the procedure. Inclusion criterion for patient recruitment was pre-menopausal status. All recruited patients were scheduled to undergo a laparoscopic procedure for benign conditions of the ovaries. Patient age ranged from 19 to 37 years (mean age 31.2 ± 6.48). Small biopsies of ovarian tissue collected from the cortex surrounding benign cysts is a good source of ovarian tissue to study cryopreservation procedure [9]. The study was reviewed and approved by the Ethics Committee of Canton Zurich (Kantonale Ethikkommission Zürich, KEK-ZH-NR: 2010-0169/0).

### Tissue preparation

For tissue harvesting, the ovarian cortex was excised intraoperatively with scissors without previous electrocoagulation. The specimens were immersed and kept in fresh basal medium composed of Leibovitz L-15 (Gibco) and 10 % Human Serum Albumin (HSA, Sage) at room temperature (RT), and transported to the laboratory within 5 min.

### Rapid freezing/thawing protocol

**Fig. 1:**
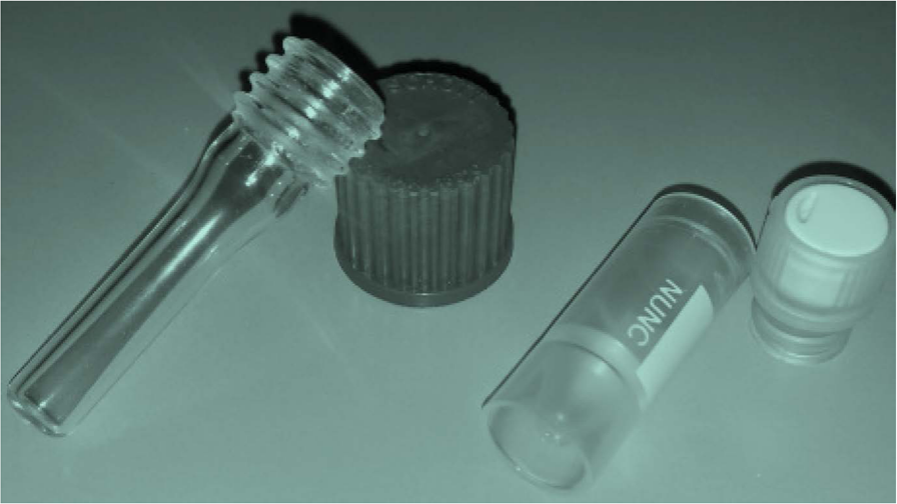
Shows the used vials: a PP vial on the right side and a quartz vial on the left side

The ovarian cortex, cleaned from the ovarian medulla, was cut into 3 thin slices, which were assigned randomly to a fresh and a cryopreserved group. One of the slices was fixed immediately and used for fresh histological analysis, and the remaining pieces were cryopreserved rapidly in three solutions with increasing cryoprotectant concentration using the following protocol. All cryoprotectants were from Sigma-Aldrich: EG, ethylene glycol; DMSO, dimethyl sulfoxide; and PROH, 1,2-propanediol. Quartz and PP vials were refilled with solution 1, solution 2, and solution 3. Then, slices were immersed in both tubes (Fig. 1) in solution 1 (1 M EG in Leibovitz L-15 supplemented with 20 % HSA) for 8 min at RT, then passed through solution 2 (1 M EG, 1 M PROH, 1 M DMSO, 0.3 M sucrose in Leibovitz L-15 supplemented with 20 % HSA) for 5 min at RT, and finally equilibrated in both kind of tubes in fresh solution 2 at 4 °C for 15 min. We decided to use a rather long equilibration at 4 °C in step 3 in order to minimize toxicity, and because DMSO and EG penetrate tissue faster at 4 °C than do PROH and glycerol (GLY), as also suggested by Newton et al. [10]. At each step, tubes were rolled gently to allow penetration of cryoprotectants. All cryoprotectant solutions were filter-sterilized and stored for a maximum of 3 days at 4 °C prior to use. After removing all additional medium using sterile absorbent gauze, tissues were placed in the inner wall of sterile pre-cooled PP vials (1.8 ml NUNC cryotube, Sigma-Aldrich; method B), or pre-cooled quartz vials sterilized (H. Baumbach & Co Ltd., UK; method C), both closed, immersed in liquid nitrogen for 30 min and then stored in vapor storage tanks (CBS V1500-AB series). After a month, samples were thawed rapidly within 10 s at RT, followed by immersion in a water bath at 38 °C for 1 min. The contents of each vial were transferred at RT into solution I (Leibovitz L-15 supplemented with 0.5 M sucrose and 20 % HSA) for 5 min, then into solution II (Leibovitz L-15 supplemented with 0.25 M sucrose and 20 % HSA) for 5 min, and finally into solution III (Leibovitz L-15 and 20 % HSA) for 5 min, washed (3–5 times), and then incubated (HERA cell 150, Thermo Scientific) for 12–15 h in G-IVF medium (Vitrolife) in 6 % CO_2_ in air at 37 °C. The vial was agitated gently throughout the thawing steps. To allow immunohistochemical (IHC) analysis, all frozen/thawed samples were incubated for 12–15 h to provide time for protein expression, but not longer than 15 h to reduce any influence of culture on our cryopreservation results.

### Use of different vials

Follicle survival depends on cryoprotectant concentration and exposure time [6]. In this context we developed the idea of testing a new cryopreservation protocol (non-equilibrium procedure), i.e., a hybrid technique in which cryoprotectant concentration is reduced with respect to vitrification using a combination of factors. Firstly, we used quartz vials instead of the more commonly used polypropylene (PP) vials. For both quartz and PP vials we used a closed system in which tissue never contacts liquid nitrogen directly, in accordance with EU Directive (http://eur-lex.europa.eu: 2004/23/EC, 2006/17/EC, 2006/86/EC). Quartz (8 Wm^−1^K^−1^) has a better conductivity than PP (0.16 Wm^−1^K^−1^) [7, 8]. Secondly, we performed the first freezing step in 1 M EG. EG has a molar mass of 62.07 g · mol^−1^ [cf. DMSO 78.13 g · mol^−1^ and PROH 76.09 g · mol^−1^). Its small mass, combined with its high permeability of 1.85 × 10^−9^ m^2^/s (cf. PROH 1.0 × 10^−9^ m^2^/s), allows EG to enter cortical tissue more easily and faster than other cryoprotectants [11].

### Histological analysis

The morphological appearance of follicles, stromal cells and vessels were examined before and after cryopreservation. Fresh and frozen/thawed ovarian tissue was fixed in 4 % formalin, washed in 70 % ethyl alcohol, and then processed and embedded in paraffin wax. Serial sections 4 μm thick were prepared and, for every eight sections, three slices were mounted on glass slides (Thermo Scientific). The central slice was deparaffinized, hydrated and stained with haematoxyilin and eosin (H&E), while the other two slices were used for IHC detection. All sections were observed using a light microscope (Olympus, model BX43) at magnifications from 25X to 400X, and scored blind after assigning a random number to each slide. All photos were taken using a light microscope (Micro Zeiss AXIO Images) connected to a camera (Axiocam MRc5 Zeiss, controlled by Axiovision Release 4.8). To help reduce costs, IHC was performed only if a follicle was found in the H&E stained slide. To avoid double counting of follicles, only follicles containing oocytes with a visible nucleus and granulosa cells (GCs) were evaluated. The developmental stage of the follicle was evaluated according to Gougeon’s criteria, and follicle morphology was evaluated according to Keros’s modified parameters [12, 13].

A score of one to three was given to each follicle. *Level 1* corresponded to well-preserved follicles that contained an intact oocyte in contact with the surrounding intact GCs. *Level 2* corresponded to quite well preserved follicles that showed no more than 25 % of one or two of the following signs: detachment of the oocyte from surrounding GCs, vacuolization of the cytoplasm, disorganized and degenerated GCs, or detachment of the basal membrane. *Level 3* corresponded to degenerated follicles that showed more than 50 % of the following signs: detachment of the oocyte from surrounding GCs, vacuolization of the cytoplasm, disorganized and degenerated GCs, detachment of the basal membrane, or having pyknotic oocyte nuclei.

Stromal cells were evaluated using a score from one to four. *Level 1* denoted very good preservation (81–100 % undamaged nuclei, no edema, no pyknotic nuclei); *level 2* denoted quite good preservation (60–80 % undamaged nuclei, rare edema, rare pyknotic cells); *level 3* denoted very poor preservation (31–59 % undamaged nuclei, focal edema, focal pyknotic nuclei); and *level 4 denoted* bad preservation (0–30 % undamaged nuclei, diffuse edema, diffuse necrotic stromal cells with pyknotic nuclei).

Histological evaluation was also undertaken to detect potential micro vascular damage, using a score from one to four: *level 1* indicated good preservation (81–100 % undamaged nuclei endothelial cells, and vascular endothelium with no sign of detachment), *level 2* indicated quite good preservation (60–80 % undamaged nuclei endothelial cells, and vascular endothelium with rare signs of detachment), *level 3* indicated very poor preservation (31–59 % undamaged nuclei endothelial cells, and vascular endothelium with focal signs of detachment), and *level 4* indicated bad preservation (0–30 % undamaged nuclei endothelial cells, and vascular endothelium with diffuse sign of detachment).

### Immunohistochemical analysis

We used semiquantitative IHC analysis to evaluate the preservation of stromal cells, follicles and vessels subjected to rapid freezing/thawing (*methods B* and *C*) compared to a fresh sample as a control (method A). Immunohistochemical evaluation was based on a modified classification according to Fabbri et al. (diffuse, patch, or focal pattern of the ICH staining) [14]. Three antibodies were analyzed: Ki67, Bcl2 and Hsp70. The Ki67 antibody (Cell Marque Lifescreen Ltd., 1:300 dilution) recognizes a nuclear structure expressed during all active phases of the cell cycle (G1, S, G2 and mitosis) except in quiescent or resting cells (G0 phase); Ki67 act as a cellular marker for cellular proliferation, and is an excellent index with which to evaluate the potential growth fraction of a tissue in research and diagnostics [15]. The Bcl2 antibody (Novocastra laboratories Ltd, 1:200 dilution) recognizes a mitochondrial membrane protein that blocks programmed cell death, and thus acts as an anti-apoptotic index. The Hsp70 antibody (Invitrogen BV, 1:400 dilution) is expressed during different kinds of stress, and acts as a stress index. Specificity of staining was confirmed by a detection-system tested for unspecific staining on human tissue, and by positive controls from a multi-tissue Array. The grade of immunohistochemical positivity was evaluated according to Fabbri’s modified parameters as: diffuse (≥ than 50 % of cells are positive), patch (20–50 % of cells are diffusely positive), and focal (<20 % of cells are positive in fewer zona).

### Statistical analysis

Data were described as numbers and percentages, or mean and standard deviation, as appropriate. Histological results were analyzed statistically. Statistical evaluation was undertaken using Intercooled Stata 11.0 (StataCorp LP, College Station, TX) by means of Fisher’s exact test for categorical data. *P*-values below 0.05 indicate statistical significance (two-sided).

## Results

### Histological results

Table 1 shows the summary of the histomorphological analysis of preservation for follicles, stromal cells, and vessels in fresh ovarian tissue, rapid frozen/thawed tissue in PP vial and in quartz vial. A total of 72 follicles at different stages were analyzed: 41.7 % at primordial stage, 16.7 % intermediary, 33.3 % primary, 5.5 % secondary, and 2.8 % pre-antral and antral stage. 58.4 % of the follicles were at the “resting stage”. Morphological analysis of the follicles revealed better preservation with method C (quartz vials) than with method B (PP vials) (92.3, and 36.4 %, respectively). Quartz vials lead to a statistically significantly higher preservation score than method B (*p* = 0.008).

**Table 1:** Histomorphological analysis of preservation for follicles, stromal cells, and vessels in fresh (A) ovarian tissue, rapid frozen/thawed tissue in PP vial (B), and in quartz vial (C)

Preservation	Score	A - fresh ovarian tissue	B - rapid frozen/thawed tissue in PP vial	C - rapid frozen/thawed tissue in quartz vial	P (Fisher’s exact test)
Follicles	1	9 (90 %)	4 (36.4 %)	12 (92.3 %)	0.004*
	2	1 (10 %)	7 (63.6 %)	1 (7.7 %)	
	3	0	0	0	
Stromal cells	1	10 (62.5 %)	13 (54.2 %)	9 (47.4 %)	0.80
	2	6 (37.5 %)	9 (37.5 %)	8 (42.1 %)	
	3	0 (0 %)	2 (8.3 %)	2 (10.5 %)	
	4	0 (0 %)	0 (0 %)	0 (0 %)	
Vessels	1	5 (38.5 %)	2 (10 %)	1 (9.1 %)	0.16
	2	6 (46.1 %)	7 (35.0 %)	6 (54.5 %)	
	3	2 (15.4 %)	5 (25.0 %)	3 (27.3 %)	
	4	0 (0 %)	6 (30.0 %)	1 (9.1 %)	

For follicle preservation, score 1 is considered as well preserved, score 2 as quite good preserved, and score 3 as degenerated follicles. For stromal cells and vessels, scores 1 + 2 are considered as well preserved, score 3 as not optimal preservation, and score 4 as degenerated stromal cells or degenerated vessels, resp

Data are presented as numbers and percentage (in brackets)

*) Method B differs significantly from C (two sided Fisher’s exact test, *p* = 0.008)

The examination of the morphology of stromal cells (score 1 and 2) revealed a comparable preservation for both methods (PP vials 91.7 %, and quartz vials 89.5 %, resp.; *p* = 0.55).

**Fig. 2:**
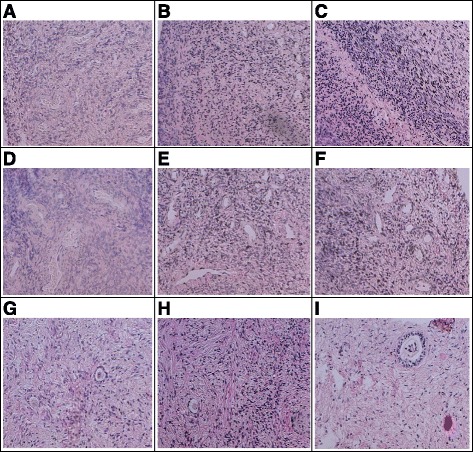
Light microscopy images of cortical human ovarian tissue stained with haematoxylin-eosin from fresh (control) tissue (**a**, **d**, **g**), cryopreserved in PP device (**b**, **e**, **h**) and cryopreserved in quartz device (**c**, **f**, **i**). Stromal cells in cryopreserved tissue using PP (**b**) or quartz (**c**) vial are good preserved and compared to fresh tissue (**a**). Vessels shows a quite good preservation using PP (**e**) or quartz (**f**), even if in PP it is observed a significant lower amount of well-preserved vessels compared to fresh tissue (**d**). It is observed in follicles a good preservation after cryopreservation in both devices, even if quartz (**i**) has a significant high good preservation compared to PP (**h**). Follicles stages show in G two primary follicles, in H one intermediary, and in I a secondary follicle. (Magnification from 100× to 400×)

Similarly, the morphology of vessels was preserved (score 1 and 2) in all cryopreserved tissue with no statistically significant differences (PP vials 45.0 %, and quartz vials 63.6 %, resp.; *p* = 0.54). Results are obtained by HE staining as shown in Fig. 2.

### Immunohistochemical results

**Fig. 3:**
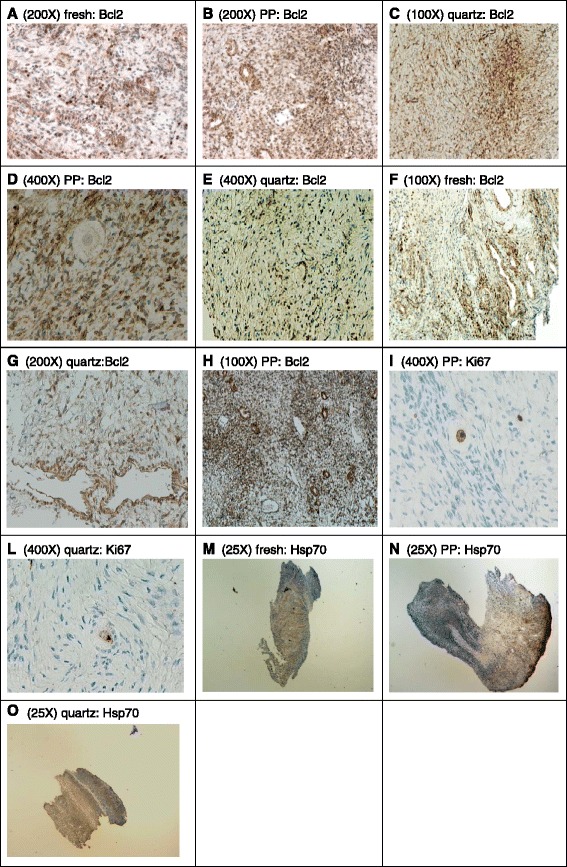
Representative images of immunohistochemical analysis of Bcl2, Ki67 and HSP70 levels in cortical human ovarian tissue. **a** microphotography shows Bcl2 staining in fresh stromal cells, compared to cryopreservation tissue in PP (**b**) and quartz (**c**). For follicles a positive Bcl2 staining was found in GCs after cryopreservation in PP (**d**) and quartz (**e**). Vessels evaluation shows a positive staining for Bcl2 both in fresh tissue (**f**) and cryopreserved tissue using PP (**h**) or quartz vial (**g**). For ki67 a positive staining after cryopreservation is shown in the nuclei like in the ooplasm using PP vial (**i**) and quartz vial (**l**). For hsp70 a diffuse positive staining in stromal cells is shows in fresh (**m**) and cryopreserved tissue using PP (**n**) or quartz devices (**o**). Cells stained positive for Bcl2, Ki67, HSP70 are brown, compared with the blue counterstained cells. Magnification from 25× to 400×

The majority of GCs showed positive staining for Bcl2 in both cryopreservation methods (B and C), with higher expression in group C (quartz vials) than in group B (PP vials) (Fig. 3). Oocytes and their nuclei showed intense positive staining for ki67 in both methods B and C (Fig. 3).

A high percentage of both stromal cells and vessels were stained for Bcl2 in method B and C (Fig. 3). In both kinds of vials, patch positive staining for Ki67 was observed in stromal cells.

Expression of hsp70 was not increased after cryopreservation, although fresh tissue showed a diffuse positivity compared to frozen tissue (Fig. 3).

## Discussion

Currently, the standard procedure for ovarian tissue cryopreservation is a slow freezing followed by rapid thawing protocol. However, the long duration (>3 h) needed to achieve the required slow reduction in temperature, and the use of a significant amount of liquid nitrogen is disadvantageous. Also, even slow freezing cannot avoid ice crystal formation with the associated negative effect on oocyte survival. In addition, many authors also report suboptimal stromal cell preservation during slow freezing [13, 16]. A disadvantage of vitrification procedures is the use of very high cryoprotectant concentrations (more than 4 M), reaching cytotoxic levels.

Our study focuses on a new, rapid cryopreservation protocol that avoids the disadvantages of slow freezing and vitrification, with the aim of achieving high recovery of all cells presented in ovarian cortical tissue as a prerequisite for optimum transplantation functionality. We evaluated a faster freezing procedure that avoids high – and thus cytotoxic – cryoprotectant concentrations. Of the cryoprotectants tested, EG has the lowest molecular mass. Another important factor in our method is that the thermal conductivity of quartz is higher than that of PP. Thus, we directly tested the effects of quartz during our rapid cryopreservation procedure. Quartz has been tested successfully in oocytes using special quartz capillaries [7, 8, 17, 18].

Both the cryoprotectant penetration rate and the temperature play important roles during the cryoprotectant procedure; a compromise between cryoprotectant concentration, incubation time and temperature is required [19].

A first freezing step using only the smallest cryoprotectant (EG) combined with a quartz vial as support resulted in more rapid penetration, decreasing both exposure time and cytotoxicity.

Our morphological results showed better preservation of follicles with Quart vials (method C) compared to PP vials (method B). Frozen/thawed stromal cells were compared to fresh controls in both methods. These histological results agree with the results of IHC, observing the distribution and relative amounts of Bcl2, Ki67, and HSP70 in human cortical tissue. Ki67 and Bcl2 were also tested by previous authors as markers for preservation during freezing/thawing procedures [14, 20, 21].

Diffuse positivity for Bcl2 in GCs, stromal cells, and vessels observed after cryopreservation in both devices confirmed that these cells are not in a state of apoptosis. In vessels, Bcl2 is also involved in production of vascular endothelium growth factor (VEGF) as reported by Sasi et al. [22]. Thus, diffuse positivity observed in vessels could indicate not only an inhibition of apoptosis but also an induction of vascular endothelium growth crucial for proper transplantation of cryopreserved ovarian tissue.

Diffuse positive staining of Ki67 in oocytes and their nuclei, and patch positive staining in stromal cells confirmed that these cells are in a growing phase and not in G0 of the cellular cycle. However, a few cells staining positive for ki67 were observed in GCs. Choi reported that ovarian tissue cryopreservation suppresses GC proliferation, and that this impairment is recovered within 48 h after culture [23]. This was confirmed in our study, with a few positive GCs staining for Ki67 after cryopreservation. This may be because tissue was fixed after 12–15 h of culture and this time is possibly insufficient for GC recovery. It might also depend on the natural disappearance of follicles during follicular growth and development, which involves primarily the apoptosis of GCs; this plays a major role in follicular atresia. Thus, only a limited number of primordial follicles develop to the pre-ovulatory stage and ovulate, the majority of follicles are eliminated throughout their normal reproductive life cycle [24, 25].

The HSP family is known to be induced in response to cellular stresses, including changes in temperature and ischemia [26]. Thus, HSP70 may be used as a bio-indicator to monitor the impact of environmental factors on an exposed organism, including on the reproductive organs [27]. However, in this study, positive Hsp70 staining was seen in both fresh and cryopreserved stromal cells, with no increased level of expression observed after cryopreservation compared to the fresh control. The expression also in fresh tissue could be due to an overexpression correlated to the pathogenesis of cystic disease [28], as all our analyzed cortical tissue came from patients affected by ovarian cysts. Alternatively, it could be due to a constitutive expression of hsp70 observed in human cells also under normal physiological conditions [29].

## Conclusion

This is the first study to test quartz property as device on ovarian tissue cryopreservation procedure. Based on these data it can be concluded that quartz is a better material for cryopreservation than PP. Using quartz vials led to larger numbers of good follicles with comparable results for both stromal cells and vessel preservation. We hypothesize that this may be because quartz material can maximize the cooling rate. These promising results suggest that this cryopreservation protocol is efficient and could be a suitable technique for ovarian tissue cryopreservation if quartz vials are used. However, further clinical studies on a larger patient group are necessary in order to evaluate follicle genetic damage after cryopreservation, and to validate functional recovery using ovarian xenografting.

## Abbreviations

DMSO: Dimethyl sulfoxide
EG: Ethylene glycol
GC: Granulosa cells
GLY: Glycerol
H&E: Haematoxylin and eosin (staining)
HSA: Human serum albumin
IHC: Immunohistochemical (analysis)
PP: Polypropylene
PROH: 1,2-propanediol
RT: Room temperature
VEGF: Vascular endothelium growth factor
